# Assessment of a medical physics educational program for science teachers

**DOI:** 10.1002/acm2.70087

**Published:** 2025-04-03

**Authors:** Ashley J. Cetnar, Jeffrey Woollard, Lin Ding

**Affiliations:** ^1^ Department of Radiation Oncology The Ohio State University Columbus Ohio USA; ^2^ Department of Teaching and Learning The Ohio State University Columbus Ohio USA

**Keywords:** education, outreach, program, teachers

## Abstract

**Introduction:**

Medical physics is a fulfilling profession where physics is applied to advance human health. However, many are uninformed of the role of physicists in medicine, and students are unaware of this career pathway. This study presents a pilot 1‐year program for science teachers to learn about physics in medicine and share with students and teachers.

**Methods:**

A cohort of middle school and high school science teachers were selected to learn about physics in medicine, develop lesson plans for their students, participate in a Physics in Medicine field trip hosted at a cancer hospital, and concluded with a professional development day for other regional science teachers. Surveys were conducted throughout the program to assess attitudes toward teaching medical physics, content knowledge of medical physics, collaboration, and demographic information from participants.

**Results:**

The program was implemented over the course of a year which included 5 school districts, 10 science teachers, and hundreds of students. After participating in the program, teacher scores on surveys regarding attitudes toward teaching medical physics and content knowledge significantly increased for the cohort. Strong collaboration between teaching pairs was maintained throughout the program based on survey responses. Teachers participating in the 1‐day professional development program also benefited from the program based on survey responses regarding attitudes toward medical physics and interest in learning more about medical physics.

**Discussion:**

This pilot study demonstrated the feasibility and effectiveness of an educational model for teachers’ understanding and connecting medical physics with students in their schools.  The program was well received by teachers and students, and this manuscript provides guidelines for effective replication of the curriculum at other institutions.

## 1 INTRODUCTION

Medical physics is a field that applies the principles and methods of physics to healthcare, focusing on the prevention, diagnosis, and treatment of diseases. Contributions of physics in medicine have revolutionized modern healthcare and is foundational for the future of medicine. Physicists have contributed many transformative devices to medicine including the invention of linear accelerators for the treatment of cancer, cyclotrons to create radioactive isotopes for clinical use, development of novel imaging technology, and intensity modulated radiation therapy for delivery of accurate and precise radiation treatments.^1^ However, when one considers professions related to advancing human health, physics is typically not the first discipline considered.

Medical physicists undergo board certification to ensure the safety and effectiveness of medical devices, optimize treatment techniques, and develop new therapeutic methods to improve patient care and outcomes. However, very few medical physicists were aware of the vocation while they were in high school, and many learned about the career through their personal networks or by happenstance later in life. Medical physics is a growing field, but the lack of awareness of the field at early‐stages may contribute to inability to meet the growing demands in the workforce.

To help promote awareness of the application of physics in healthcare, a program was developed partnering medical physicists with grades 6–12 educators to explore how physics is applied in the diagnosis and treatment of cancer as an interdisciplinary project. Medical physicists, education researchers, and science teachers collaboratively developed lesson plans and educational content. The goal of this program was to strategically engage with the next generation through education to increase the awareness of the meaningful opportunities for physicists to contribute to healthcare.

The purpose of this study is to report on the program design and implementation and assess the impact of an educational training program by assessing teacher participants’ attitudes toward teaching medical physics, medical physics content knowledge, teaching strategies for instructional practice, and the level of interdisciplinary collaboration throughout the program. The impact of the program is based on teacher's new content matter knowledge related to medical physics and self‐assessment of collaboration with other science teachers.

## 2 THEORETICAL FRAMEWORK

High school and middle school teachers are formative influences in the trajectory of the future careers of the next generation. A year‐long program for grades 6–12 science teachers provided an opportunity for 10 teachers in a cohort to learn about physics in medicine. This project was built on the theoretical framework of Vygotsky's Sociocultural Theory,^2^ including the idea of the Zone of Proximal Development, which is the space between what learners can do independently and what learners can achieve with the guidance and direction of a skilled facilitator. The year‐long program for teachers was uniquely designed to enable collaborations between teachers and subject matter experts on the application of physics in medicine. This provided the opportunity for teachers to explore areas of physics research that would not be accessible on their own and partner them with content experts to reach students searching for how they can apply and develop their talents. By interacting with subject matter experts and peers, teachers were provided an opportunity to master new content beyond their individual abilities to promote growth.

The teachers’ development of lesson plans for their students facilitated the extension of the zone of proximal development to the students within the region in a way that could not have been done by medical physics content experts alone. From the student perspective, most middle school and high school students would not be able to develop the awareness or understandings of physics application in medical sciences by their own. Providing training for science teachers by medical physicists can increase the number of students reached by the education while optimizing the time and resources needed from medical physicists. After the development of the content through a shared language of teachers and students, these lessons were presented to a broader audience of science educators through a professional development day to increase the number of teachers who were trained about medical physics. The lessons were shared online for broad dissemination for science teachers wanting to access and implement medical physics‐related lessons in their classrooms.

## 3 METHODS

### 3.1 Program description

**FIGURE 1:**
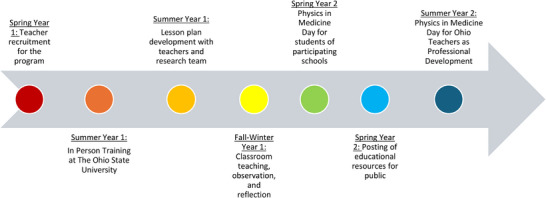
Timeline for events for the medical physics education program for science teachers.

A summary of the timeline for the educational program is shown in Figure 1. This includes recruitment, on‐site training, lesson plan development and classroom teaching, physics in medicine day field trip, posting of lesson plans online, and the professional development day open to all science teachers.

#### 3.1.1 Recruitment

Physical science and life science teachers from the same school were recruited so the science could be taught in a way that was not siloed or compartmentalized and to promote collaborative teaching. The interdisciplinary nature of the teacher assignment between two scientific domains was important for developing cross‐disciplinary relationships within the same institution to foster future collaboration within the individual school. By bringing groups of teachers together within the region to participate in the program, a network of collaboration was promoted within teacher participants.

**FIGURE 2:**
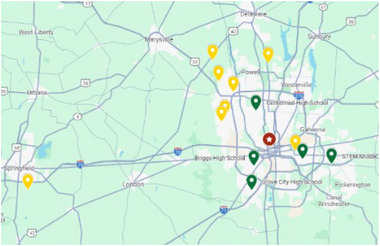
Image of applicant schools for program: applied (yellow) and accepted (green).

The recruitment of teachers for the program took place from applicants teaching physical science and life science from middle schools and high schools in central Ohio shown in Figure 2. Teachers invited to the program could earn a stipend up to $4000 for completing all four major components–onsite training, lesson plan development, student field trip, and professional development day–of the year‐long program. Completed applications from 34 teachers were received and reviewed. Specific target schools for this project included high‐needs schools and those serving underrepresented populations to provide this opportunity for collaboration. A rubric was developed for scoring applications with three reviewers. Ten teachers, in pairs from five different schools, who indicated a desire for interdisciplinary collaboration and had record of teaching excellence as program ambassadors were invited to participate in the year‐long program. Eight teachers were selected from high schools and two teachers were selected from a middle school.

#### 3.1.2 On‐site training

**FIGURE 3:**
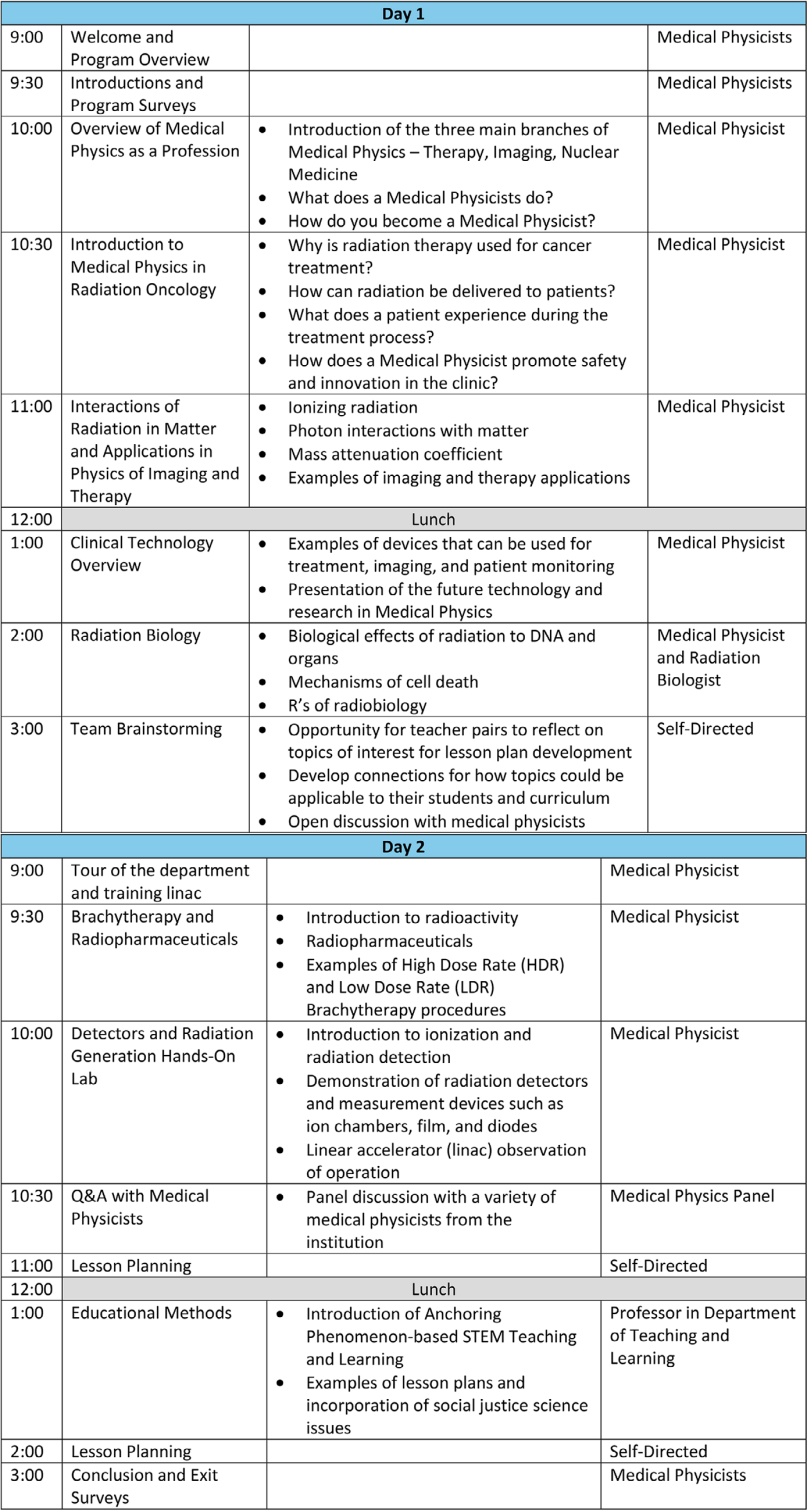
Example of 2‐day schedule that was hosted on‐site for the teachers at The Ohio State University.

**FIGURE 4:**
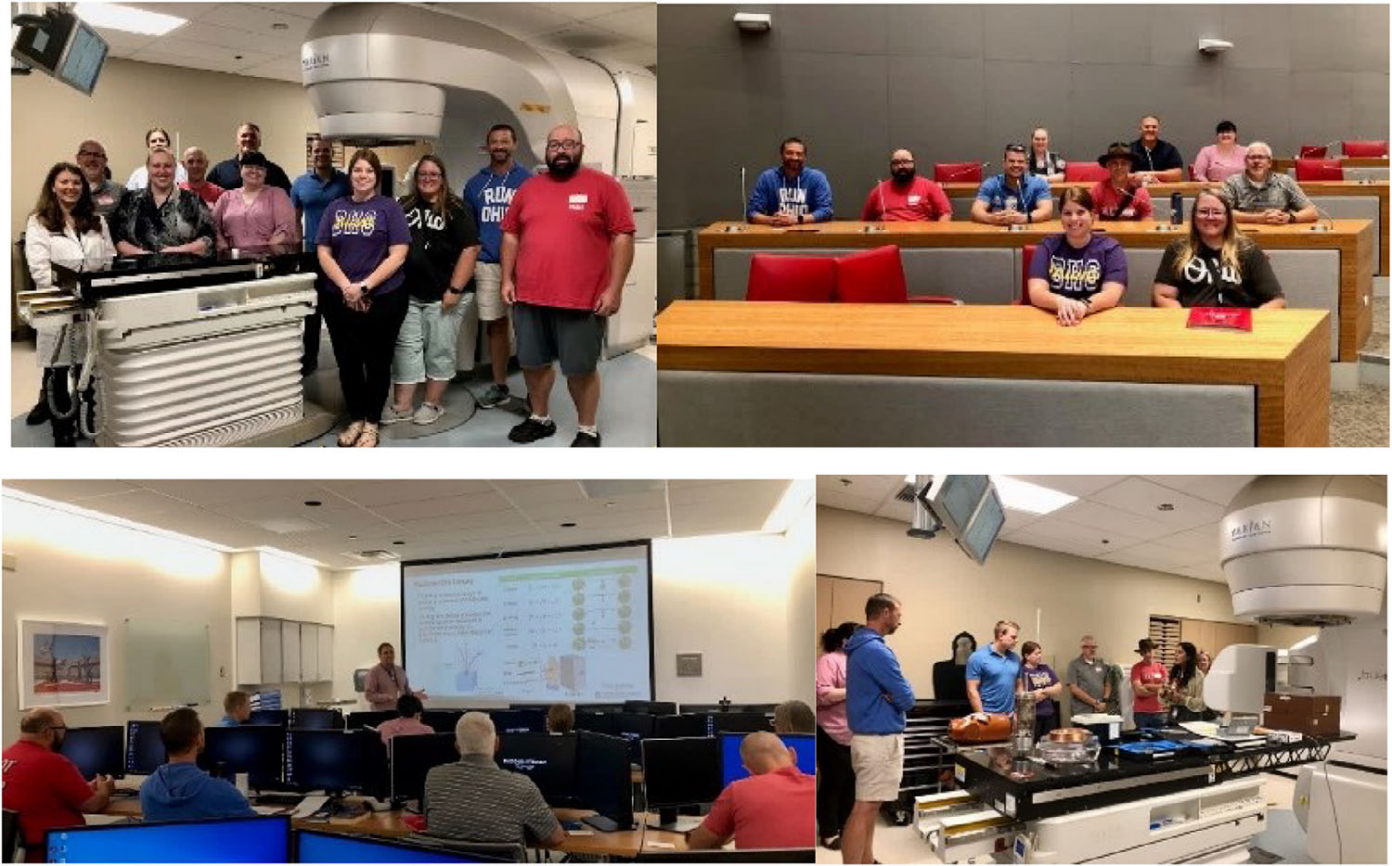
Teachers learned by traditional lecture and active learning methods to encourage questioning and understanding of the new field. Participants were selected to represent physics and biology from their school districts to promote interdisciplinary collaboration. Teachers engaged in hands‐on learning experiences where they were able to apply their didactic knowledge gained in presentations through demonstration to connect theory with application using materials from the clinic including detectors used clinically and example immobilization devices.

During the summer, teachers were invited to a 2‐day hands‐on training to learn what it is like to be a medical physicist at the cancer hospital. Teachers were able to explore the physics content through a series of presentations and practical, hands‐on activities. The sessions included a tour of the Radiation Oncology Department, introduction to medical imaging and radiation therapy delivery using a non‐clinical linear accelerator, and discussions with medical physicists. Teachers participated in reflection opportunities for the application of physics in medicine throughout the sessions. Specific scheduling and topical information can be found in Figure 3. Teachers participating in the program within the sessions are shown in Figure 4.

#### 3.1.3 Lesson plan development and classroom teaching

After the in‐person, onsite training, teachers collaborated with medical physicists to begin developing lesson plans on a particular aspect of medical physics which was age and content appropriate for their students. The lessons were codeveloped among the teachers to incorporate active learning strategies and accurate scientific content in a way that is engaging for students. Teachers selected their topics of focus for the lesson plan and developed an outline for the session during the onsite training. The teachers were then paired with a medical physicist to further develop the ideas for the class and serve as a subject matter expert in the technical components of the material. This lesson plan development aligned with the zone of proximal development connecting medical physicist subject matter knowledge with the knowledge of the high school and middle school teachers as experts in education for their students. The social cultural framework was supported by interdisciplinary peer development of the lesson plans.

Virtual implementation sessions were offered and additional correspondence via email was conducted with teachers to support application of lesson plans throughout the fall semester. Teachers taught the lesson with their students in either the fall or winter semester based on institutional scheduling constraints. Examples of topics developed for students included radioactivity in medicine, physics of imaging, biology of normal tissues, radiation vault design for pediatric patients, and radiation case studies. Many also included information about medical physics as a profession to students. Final lessons ranged from a single class period to multi‐week units incorporating collaborative teaching between the physics and biology teachers within the schools. After implementation, a large‐group virtual follow‐up session was held to debrief with the teachers and lesson plans were refined before the physics in medicine day field trip. These sessions were held virtually to increase accessibility for participants during the school year.

#### 3.1.4 Physics in medicine day field trip and posting of lesson plans

Physics in medicine day was an experience unique to the students of the teachers participating in the fellowship program. Each participating school was able to invite between 10 and 20 students to the event, totaling 70 middle school and high school students with parental/guardian consent. The field trip was hosted at the Ohio State University at the James Cancer Hospital showcasing the lessons developed by all the teachers in the program, including hands‐on activities and a tour of medical equipment used for cancer treatments, so that students were able to learn about physics in a way that was directly relevant to their lives. The students visited the hospital on a weekday in February. Teachers participating in the program presented their lessons to the students in small groups.

**FIGURE 5:**
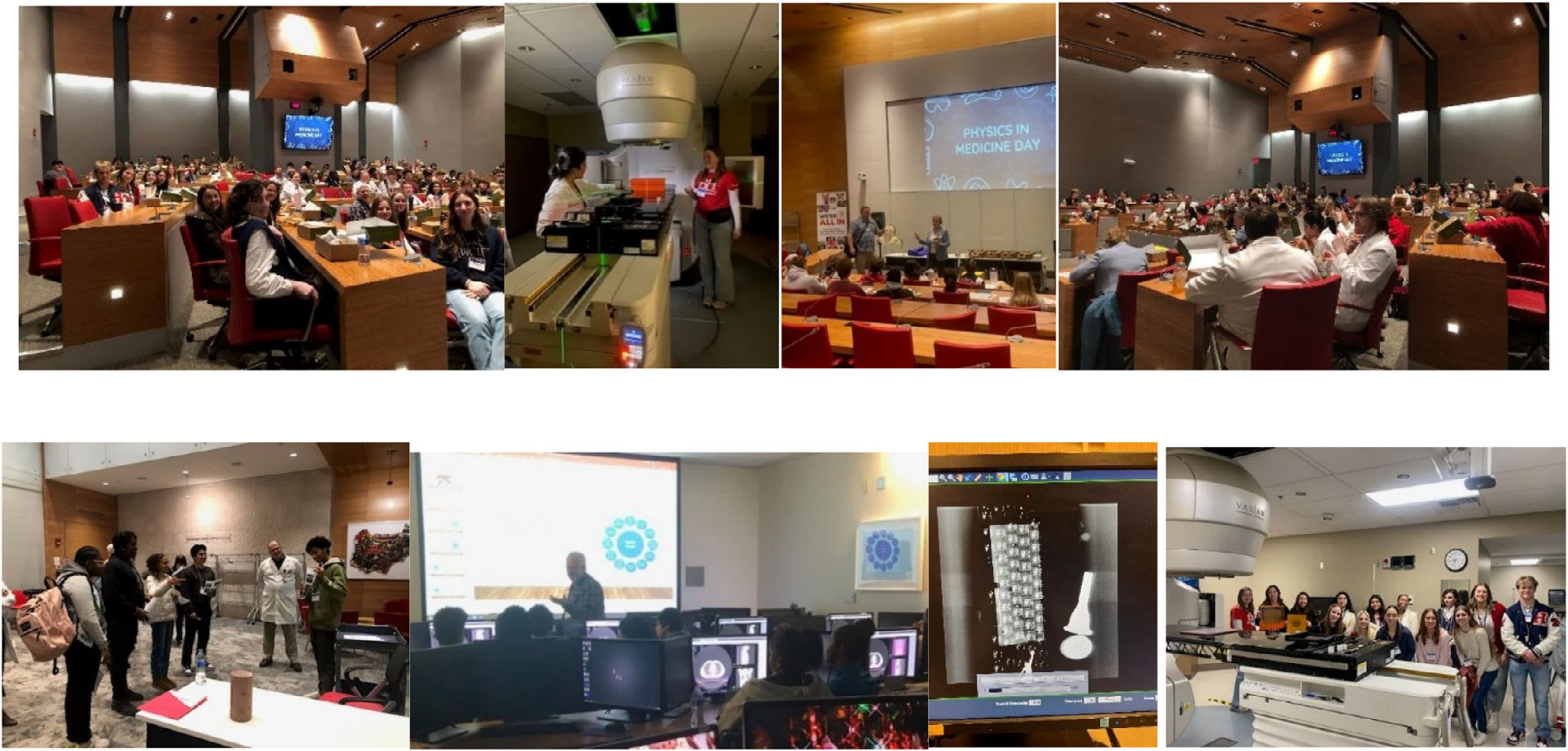
Students participating in physics in medicine day and pictures of “What's in the Box?” X‐rays for student shoe boxes.

Students observed the application of physics in state‐of‐the‐art medical equipment and experienced simulations of clinical workflows involving medical physics. One of the activities that students prepared was a shoebox from each school that was filled with items that would be imaged using a cone beam CT that is used for patient localization during radiation therapy treatment. The “What's in the Box” images from each school were displayed at the end of the day in the large group session where other schools were able to guess what the items were. Pictures of the event can be found in Figure 5. Final lesson plans were posted online so they are publicly available to facilitate learning and collaboration to advance the visibility of medical physics careers. These lesson plans can be accessed at **tinyurl.com/MedPhysforHS**. Providing the opportunity for students to learn from other high school and middle school teachers with medical physicists in a conference setting situated within a cancer hospital were designed in alignment with Vygotsky's Sociocultural Theory to maximize the opportunities for students to learn the application of physics in medicine which otherwise would not be achieved without the content experts.

#### 3.1.5 Professional development day for science teachers

**FIGURE 6:**
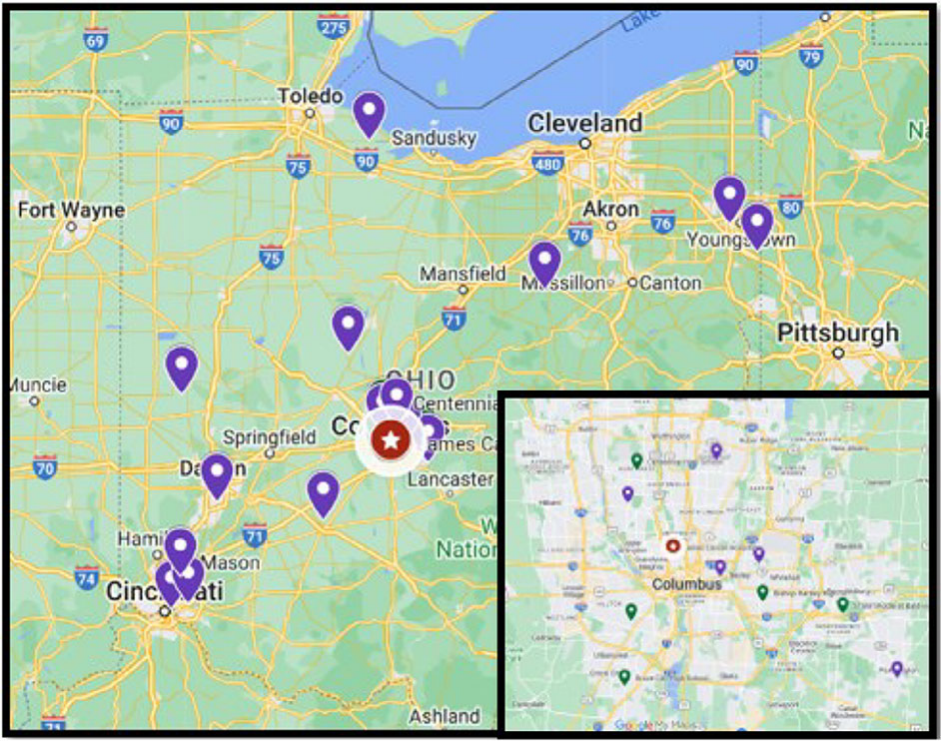
Map of registrants for physics in medicine day professional development event for science teachers. Participants (purple) and teachers who were year‐long grant awardees (green).

**FIGURE 7:**
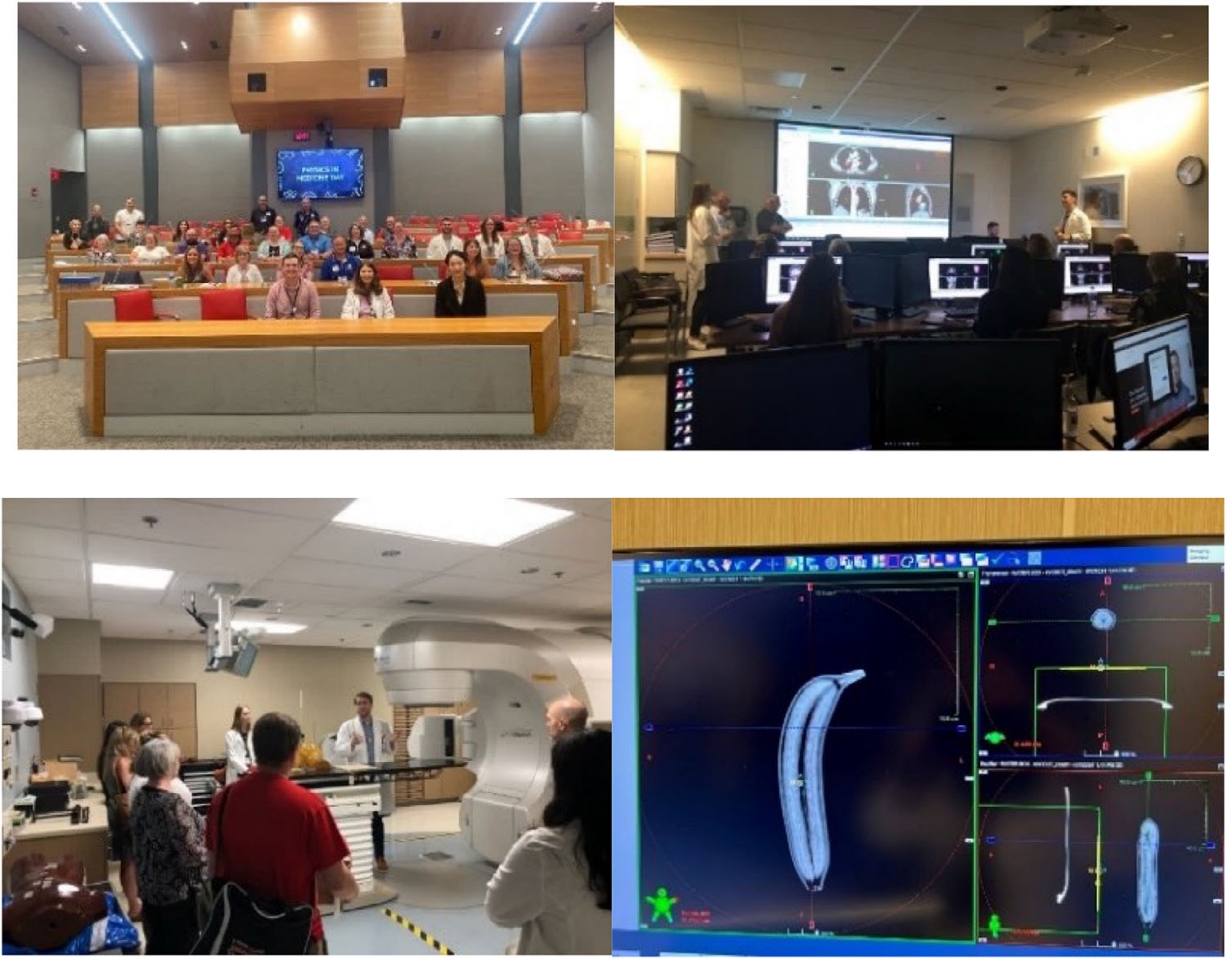
Picture of participants in the professional development day (top), participants in learning sessions (middle), and picture of “What's in the box?”.

To increase the impact of the program, a 1‐day in‐service was hosted for science teachers throughout central Ohio. Twenty‐one teachers registered for the event with the addition of the 10 teachers participating in the year‐long program. A map of registrants for the initial year‐long program and for the professional development event and pictures from the event can be found in Figures 6. The program included modeling of the teaching from the Physics in Medicine Day and additional educational programing for science teachers to engage with the application of physics in medicine in a practical way, including hands‐on activities and tours of the cancer facilities. The program was designed to optimize teacher engagement by learning from peer experts and medical physics content matter experts to increase the impact of the program for more science teachers (Figure 7).

### 3.2 Program evaluation

#### 3.2.1 Research design

The program was evaluated using surveys for teacher participants in the program, which was determined exempt by The Ohio State University Institutional Review Board (2023E0749). All teachers in the program (*N* = 10) were invited to participate in the study. The survey was designed based on several validated surveys examining the construct of collaboration within their current work environments, perceived values of collaboration and implementation of instructional practices, and interactions with their co‐teacher from their school within the program.^3^, ^4^, ^5^ The full survey included attitudes toward teaching medical physics, content knowledge of medical physics, collaboration, and demographic information from participants. Content matter questions were developed by medical physicists based on the content of the program using a 20‐question multiple choice assessment including basic physics concepts. The reliability of the assessment, measured by Cronbach's alpha, was found to be *α* = 0.823 for the attitudes toward teaching medical physics and *α* = 0.650 for the content knowledge of medical physics, both at a sufficient level of internal consistency for the items on the assessment. Results of Cronbach's alpha range from 1 to 0, where values closer to 1 indicate items on the assessment have a higher covariance or a higher agreement, meaning the measurement results are more reliable.

**FIGURE 8:**
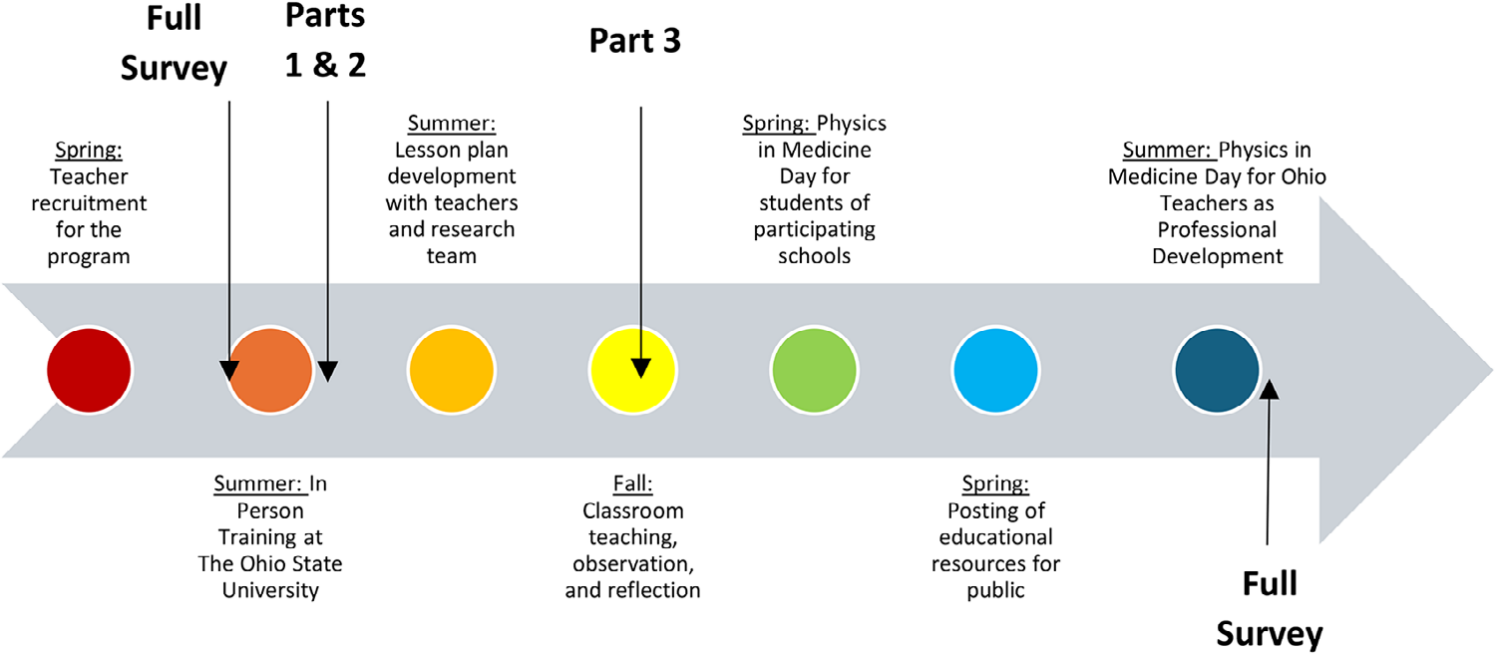
Survey timeline for distribution of full survey as a pre‐test and post‐test at the beginning and end of the program, with attitudes toward teaching medical physics and content knowledge assessed at the conclusion of the 2‐day onsite training, and collaboration between teachers during the fall semester as additional timepoints for data collection.

Surveys were conducted at four time points within the year long program shown in Figure 8. The initial survey took place on the first day of the program conducted on‐site prior to the hands‐on training as a pre‐test for baseline data for participants. The second time point was after the 2‐day on‐site training focusing on attitudes toward teaching medical physics and content matter knowledge. The third survey point took place in the fall when planning for the joint teaching session evaluating attitudes and experiences with collaboration. At the conclusion of the program there was a final exit survey after the professional development day for science teachers which included the full survey with the exclusion of the demographic information, and an open response section was included for final comments about the program regarding strengths, areas for improvement and whether they would recommend the program to a colleague.

Gains and normalized gains were calculated for the cohort. Nine of the 10 teachers completed all three evaluation sets. The results were not normally distributed for the medical physics content matter, so the Wilcoxon Signed‐Rank Test was used to statistically analyze the results with *p*‐value < 0.05 designated as the level of significance.

Teachers from the year‐long program were able to present their lesson plans a final time for their peer science teachers in a professional development day at the end of the program. Science teachers participating in the 1‐day session were invited to participate in the same attitudes survey as the teachers in the year‐long program with the addition of 5 yes/no questions. The questions included: (1) Did you hear of medical physics as a career prior to this program? (2) Would you be interested in learning more about medical physics topics? (3) Would you be interested in teaching medical physics in your curriculum? (4) Would you recommend participating in this 1‐day program to other teachers? (5) Would you participate in a 1‐year cohort as a participant in the program in the future if offered? Paired pre‐ and post‐test responses were collected from 15 of the session participants.

Surveys were printed and distributed to participants in the program to be completed in‐person at the same time in a proctored classroom setting. Data was transcribed into spreadsheets by the researchers in the study for analysis and reporting.

## 4 RESULTS

### 4.1 Teachers participating in year‐long program

#### 4.1.1 Demographic information for teacher cohort

Participants in the program were all teachers from central Ohio. The average number of years of teaching experience were 15 ± 9 years. For those who designated physical science as their current primary area of teaching the average years of experience teaching physical science was 12 ± 11, for life science was 12 ± 7 years, and middle school science 9 ± 2 years. Many of the teachers had experience teaching both physical and biological science. Within the group 9 of 10 had a master's degree as their highest education completed. Five male and 5 female teachers were members of the cohort.

#### 4.1.2 Attitudes toward teaching medical physics

**FIGURE 9:**
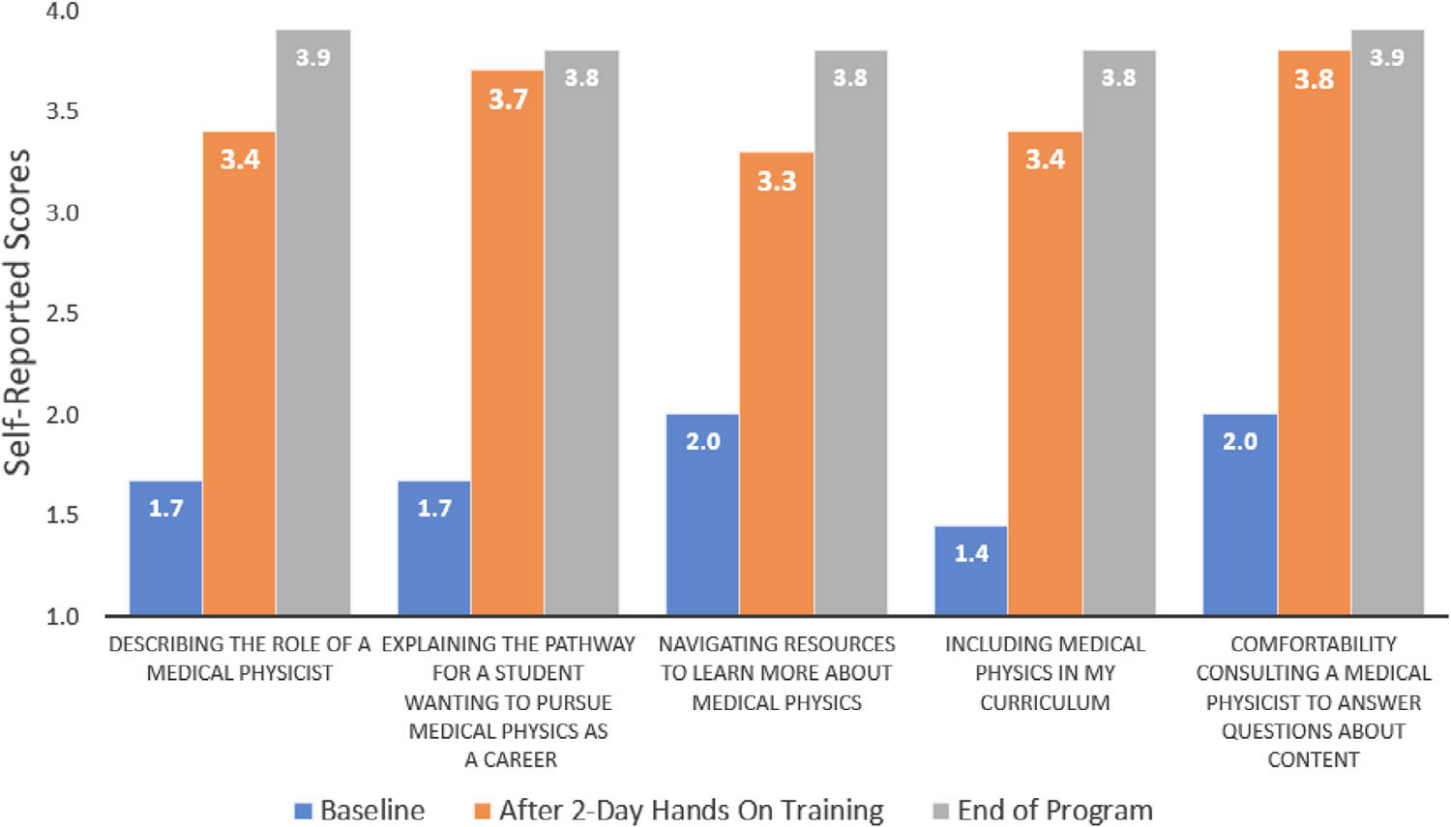
Average responses from teachers (*N* = 10) in the year‐long program in comfort for questions related to teaching medical physics where 1 represents “Very uncomfortable” and 4 “Very comfortable”.

Five questions were asked in the survey regarding attitudes toward teaching medical physics in their classrooms on a 4‐point Likert Scale where 1 represented “Very uncomfortable”, 2 represented “Uncomfortable”, 3 represented “Comfortable”, and 4 represented “Very comfortable”. Average responses in all the categories were in the uncomfortable to very uncomfortable range prior to the program as shown in Figure 9. After the 2‐day onsite training scores increased after the initial training and continued to increase with greatest confidence reported at the end of the program in all categories.

#### 4.1.3 Medical physics content knowledge

**FIGURE 10:**
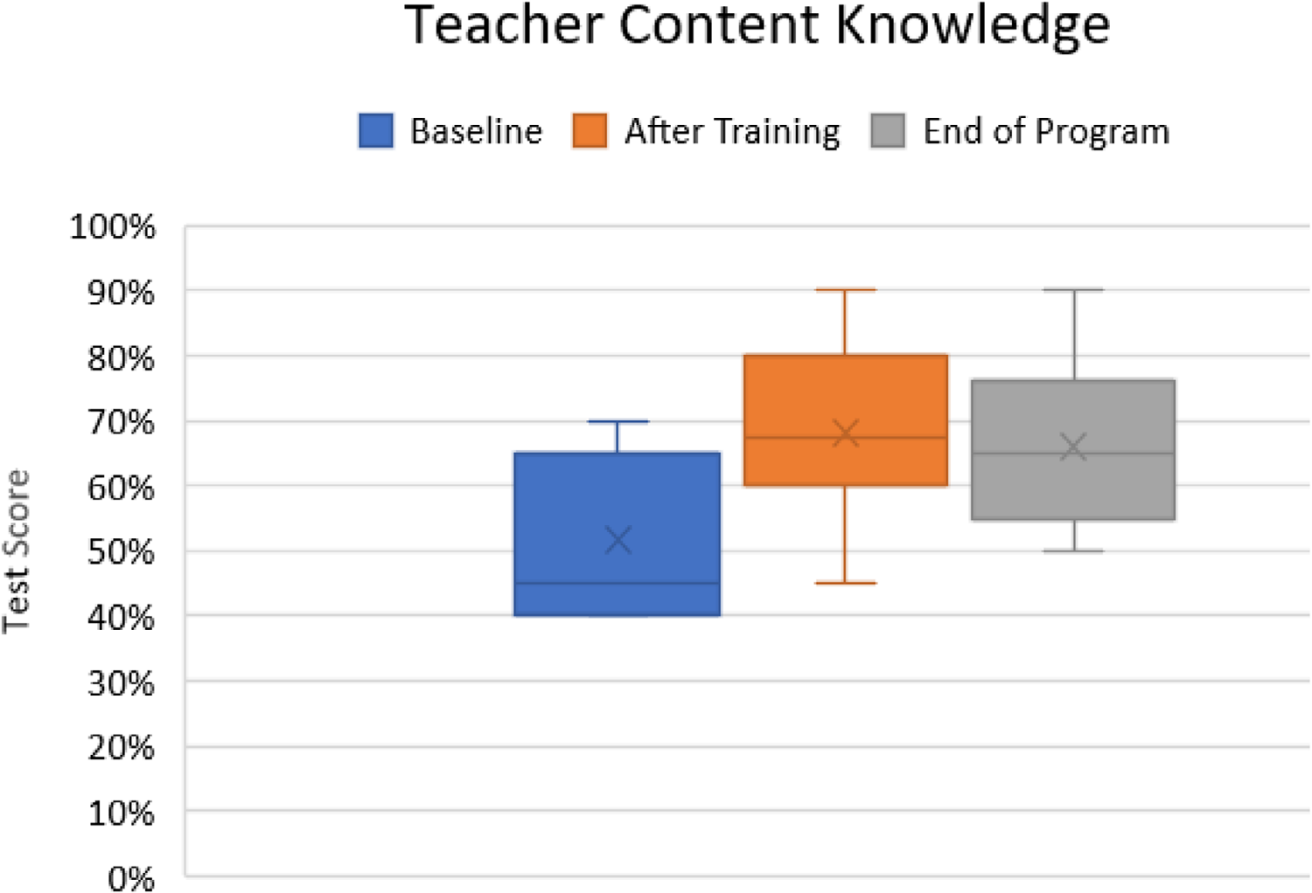
Teacher medical physics content knowledge throughout the program.

Medical physics content knowledge was assessed using a multiple‐choice test developed by medical physicists regarding content that would be presented during the on‐site training as an introduction to medical physics. Nine of the 10 teachers completed all three medical physics content knowledge assessments. Average test scores were 52% ± 13% prior to formal training and increased to 68% ± 15% after the on‐site training and was 66% ± 12% at the conclusion of the program shown in Figure 10. Results of the Wilcoxon Signed‐Rank test indicated that there is a significant difference between baseline results (Mdn = 45, *n* = 9) and after on‐site training (Mdn = 70, *n* = 9), *Z* = 2.6, *p* = 0.009, *r* = 0.9. Results of the Wilcoxon Signed‐Rank test indicated that there is also a significant difference between baseline and end‐of‐program content knowledge, *Z* = 2, *p* = 0.041, *r* = 0.7. Results of the Wilcoxon Signed‐Rank test indicated that there is a non‐significant difference between the onsite training and end‐of‐program results (Mdn = 65, *n* = 9), *Z* = −1.3, *p* = 0.203, *r* = −0.5. The normalized gain between baseline testing and the end of the 2‐day training was 33% and between baseline testing and the end of the program was 29% for the same test.

#### 4.1.4 Teaching strategies

**FIGURE 11:**
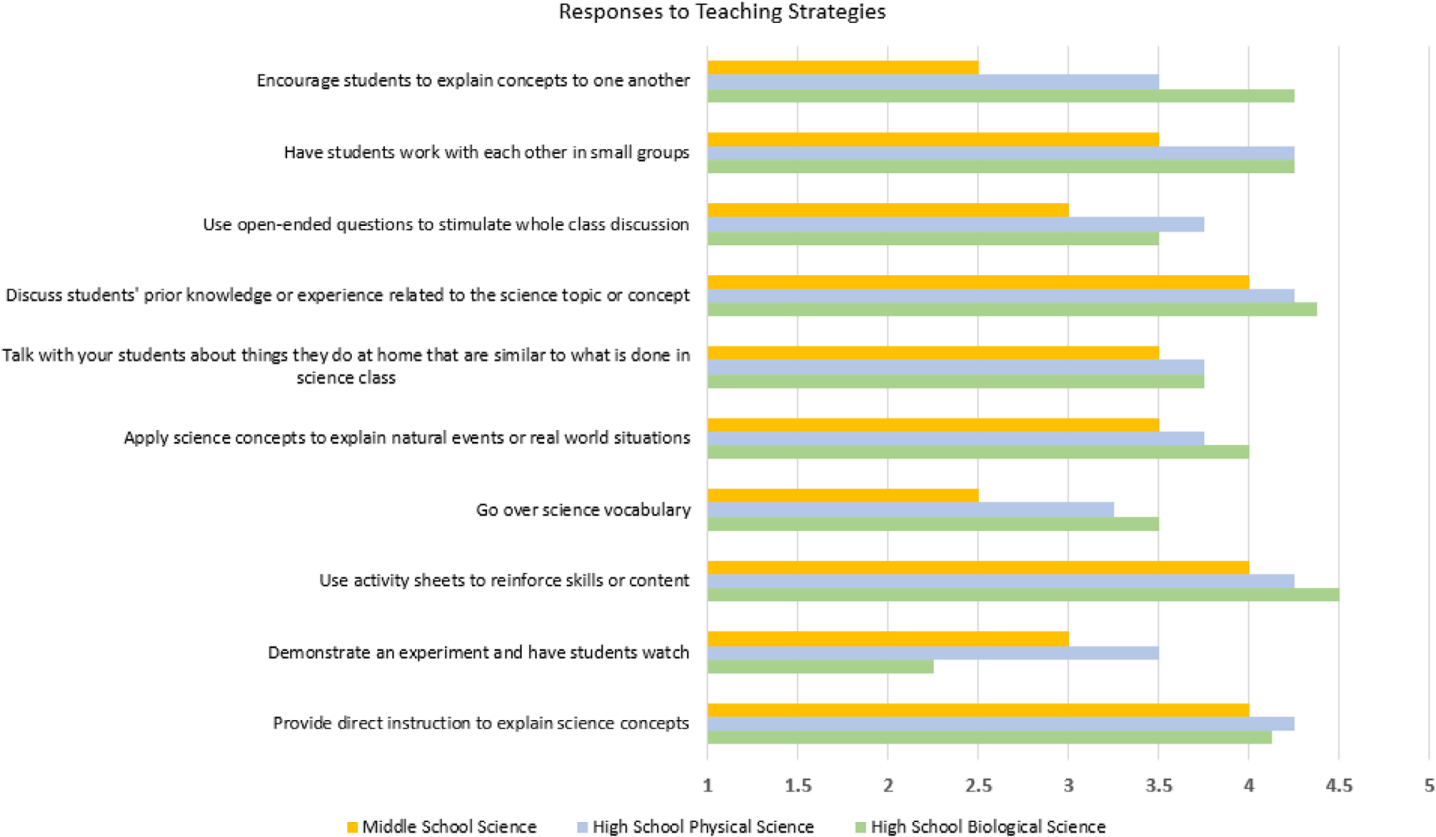
Teaching instructional methods between disciplines.

Teachers were asked in the initial survey at the beginning of the program to reflect upon their teaching strategies used during science instruction. The Science Instructional Practice Survey (SIPS) is a Likert‐scale survey with categories ranging from 1 to 5 with never (1), rarely—a few times a year (2), sometimes—once or twice a month (3), often—once or twice a week (4), or daily or almost daily (5). The results are shown in Figure 11 stratified by teachers of high school biological science (*n* = 4), high school physical science (*n* = 4), and middle school science (*n* = 2). While there are similarities in responses between most of the disciplines, the greatest differences are observed with demonstrating experiments and encouraging students to explain concepts to one another.

#### 4.1.5 Science teacher collaboration

**FIGURE 12:**
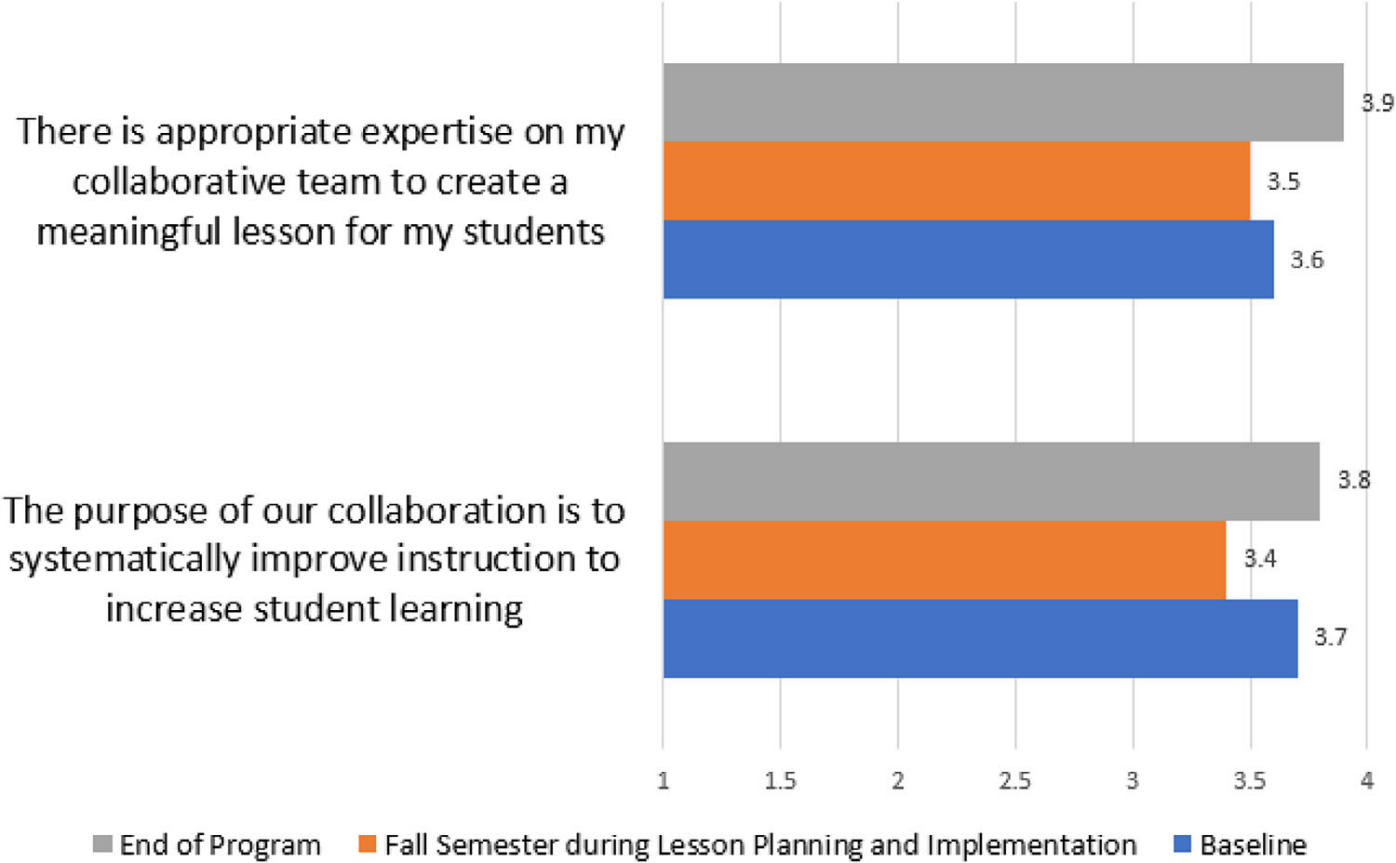
Multidisciplinary collaboration between teacher pairs within the school.

A unique aspect of the program was the pairing of teachers from the same school to promote collaboration between individuals. While strong relationships among science teachers of different disciplines may already exist within schools, this was not assumed for this study. Survey results included reflecting upon the value of collaboration within their school district and experience collaborating with their co‐teacher in the program. Teachers responded to questions on a Likert scale with strongly disagree (1), disagree (2), agree (3), and strongly agree (4). Results regarding collaboration with peer teacher are shown in Figure 12 with strong alignment with purpose and expertise within the pairs for each school throughout the program.

#### 4.1.6 Open response feedback

The final aspect of the end of program survey included an opportunity for open response feedback for the program. Teacher feedback from the year‐long program was positive, with comments highlighting the strength of the knowledge of the medical physicists, integration of science teachers with medical physicists, understanding of cancer‐related careers, applications of physics in medicine, and organization of the program. Suggestions for improvement were to continue the program for more teachers in the future, create more lessons for science teachers, and expand the amount of time the teachers can learn from medical physicists. All the program participants indicated they would recommend the program to a colleague.

## 5 TEACHERS PARTICIPATING IN ONE‐DAY PROFESSIONAL DEVELOPMENT EVENT

**FIGURE 13:**
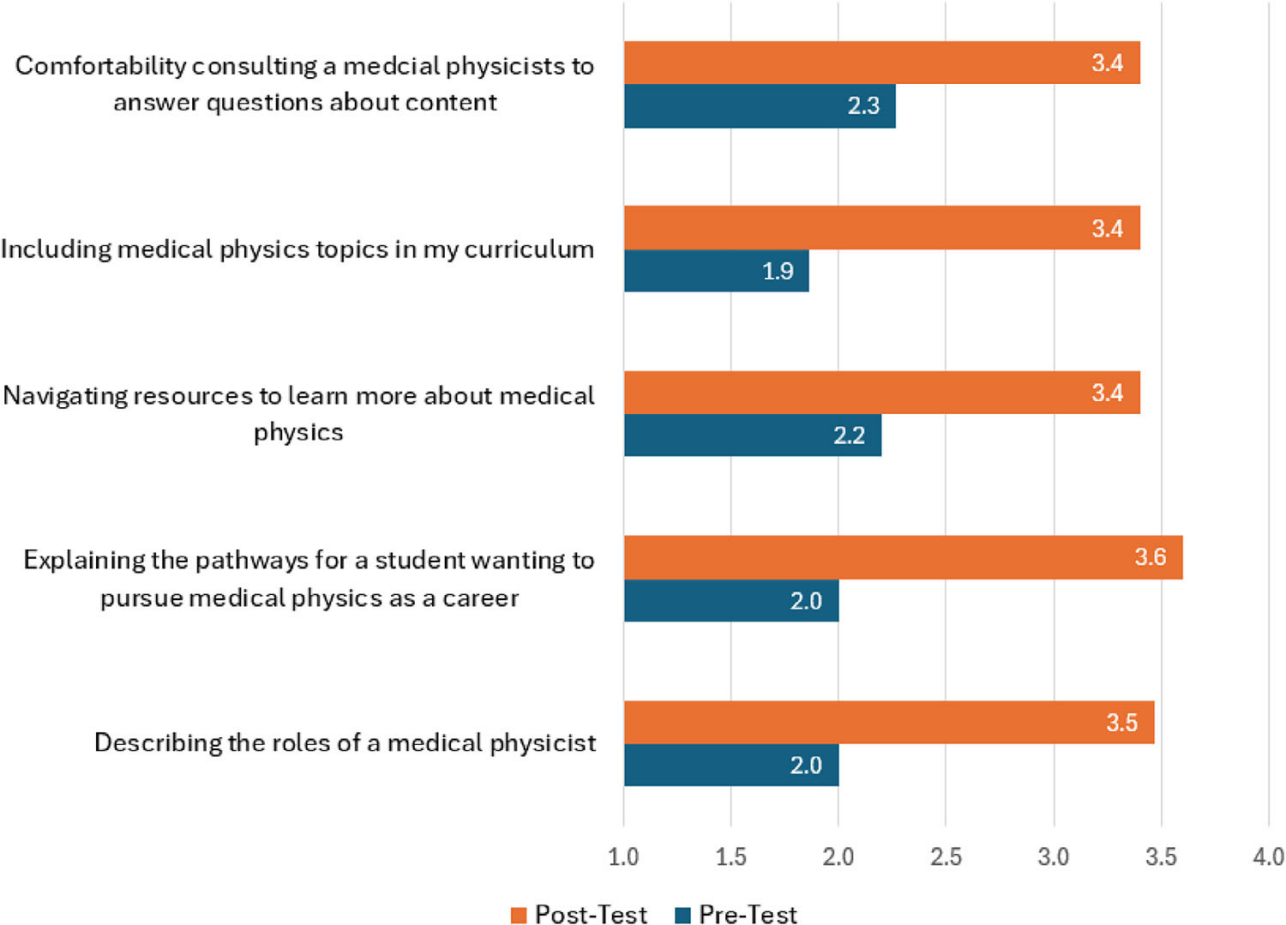
Average responses from teachers attending the professional development day teachers (*N* = 15) in comfort for questions related to teaching medical physics where 1 represents “Very uncomfortable” and 4 “Very comfortable”.

Likert‐scale questions were asked of the science teachers in the 1‐day professional program regarding teaching medical physics, scored from 1–4 where 1 represents “Very uncomfortable” and 4 represents “Very comfortable”. Before the program, mean values for the questions shown in Figure 13 ranged from 1.9 to 2.3 representing “Uncomfortable” and increased after the session to 3.4–3.6 representing “Comfortable” or “Very comfortable” for the topics regarding medical physics.

**FIGURE 14:**
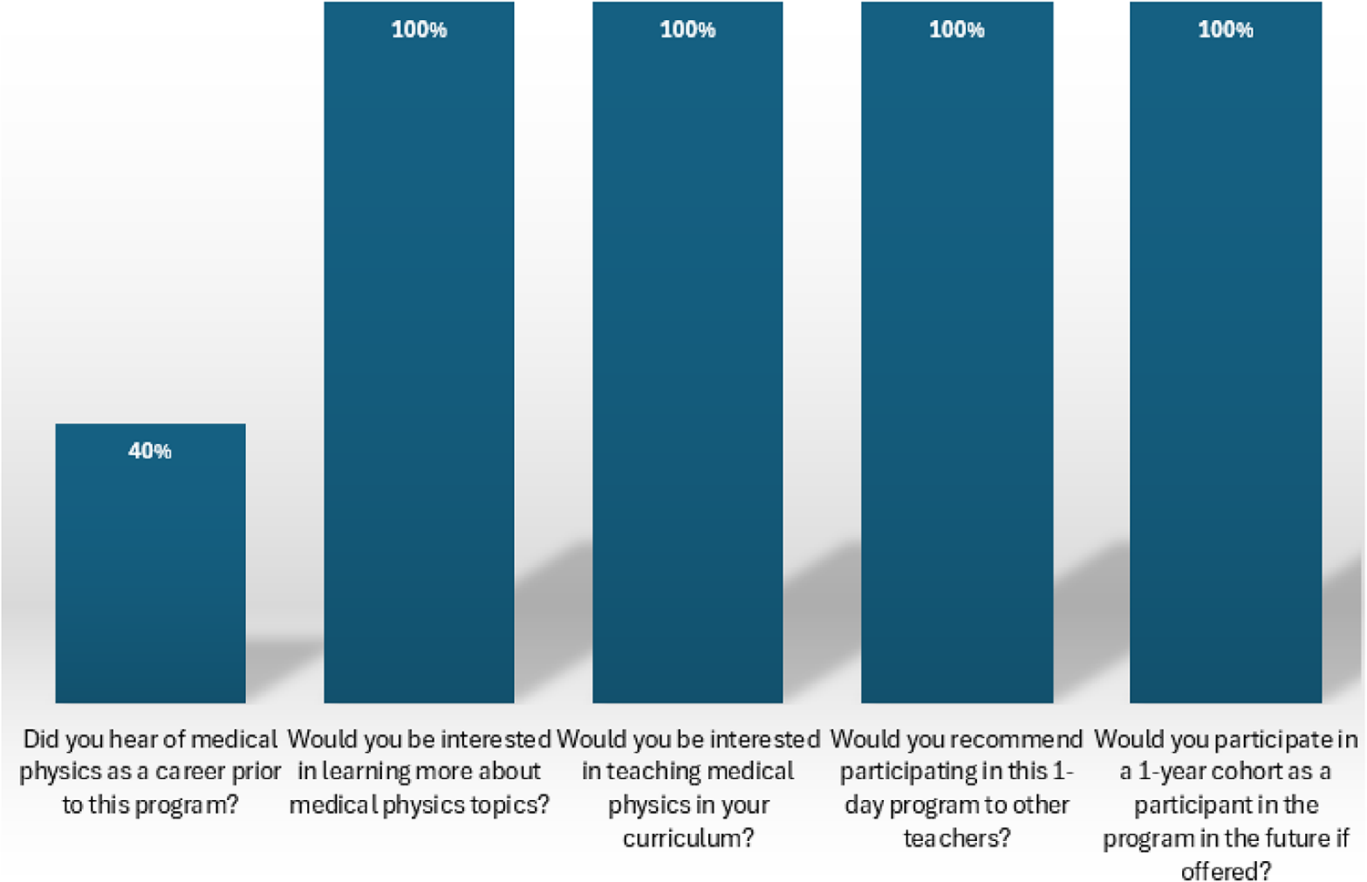
Responses from teachers in the 1‐day professional development program in comfort for questions related to teaching medical physics where 1 represents “Very uncomfortable” and 4 “Very comfortable”.

Figure 14 shows the responses from teachers in the 1‐day professional development program in comfort for questions related to teaching medical physics where 1 represents “Very uncomfortable” and 4 “Very comfortable.” Only 40% of teachers had heard of medical physics as a profession prior to the program, and 100% of participants would be interested in participating in the 1‐year science teacher training program if offered and would recommend the program to other science teachers.

## 6 DISCUSSION

The data from this program showed the value of the program in terms of increasing knowledge and comfort of high school and middle school science teachers. While other studies have provided advances in the training of students directly^6^, ^7^, ^8^, ^9^ or have developed medical physics related curriculum,^10^ this is the first project to focus on providing experiential learning about medical physics directly for science educators in high schools and middle schools. This program provided a framework for collaboration between physical and life sciences and allowed teachers to learn and create new lesson plans that could be used in their classrooms which are now publicly accessible at tinyurl.com/MedPhysforHS. This program can serve as a model for other medical institutions who wish to increase outreach efforts to provide information about physics in medicine.

This program was funded by a grant which included expenses such as educational supplies and stipends for teachers to participate in the program. The program could not have been successful without the enthusiasm and support from faculty and staff in the department and hospital leadership. While bringing visitors into the hospital can be an administrative challenge, incorporating students as minors for an educational event included additional logical considerations. Early and frequent communication and support from leadership and administration are key for successfully hosting outreach events for high school and middle school students.

While the results of the study are promising, they are limited as a single institution pilot study that was hosted for 1 year. We observed similar results in changes in self‐reported understanding and attitudes toward medical physics for both cohorts after the 2‐day training for the year‐long participants and after the 1‐day professional development day for science teachers. Additional data collection at future time points would be insightful for future studies for assessing long term outcomes of the program. While many of the survey items were adapted from validated assessments, the medical physics knowledge multiple‐choice assessment yet needs to be further validated. The reliability of the assessment *α* = 0.650 suggests a potential for the assessment to be refined in future work to ensure the validity and reliability of the measurement. The addition of having more complex questions with open‐answers and the ability to provide more context for understanding could be valuable since many of the items included in this assessment were knowledge‐based and factual in nature which may not reflect the extent of the value of the content knowledge gained in the program.

## 7 CONCLUSION

Hosting this program for presenting medical physics to science teachers was a valuable opportunity for medical physicists, science teachers, and students. Physical science and life science teachers from the same school were recruited so that the science can be taught in a way that is not siloed or compartmentalized and promote collaborative teaching. This model is presented as an effective way for teachers to connect subject matter and interact with applied interdisciplinary science. A summary of the pilot program experience shows the feasibility of this educational model. The program for presenting physics in medicine to science teachers was implemented over the course of a year which included five school districts, 10 science teachers, and hundreds of students. The program was well received by teachers and students and curriculum could be replicated for other university hospitals for medical physics outreach.

## AUTHOR CONTRIBUTIONS

**Ashley J. Cetnar**: Conceptualization; methodology; investigation; data curation; visualization; formal analysis; writing—original draft. **Jeffrey Woollard**: Conceptualization; methodology; writing—review & editing. **Lin Ding**: Conceptualization; methodology; writing—review & editing.

## CONFLICT OF INTEREST STATEMENT

Funding for the program was made possible by the Battelle Engineering, Technology, and Human Affairs Endowment.

## Data Availability

The data that support the findings of this study are available from the corresponding author, AJC, upon reasonable request.
